# ^68^Ga-EMP-100 PET/CT—a novel method for non-invasive assessment of c-MET expression in non-small cell lung cancer

**DOI:** 10.1007/s00259-022-05995-3

**Published:** 2022-10-18

**Authors:** Lena M. Unterrainer, Andrei Todica, Leonie Beyer, Matthias Brendel, Adrien Holzgreve, Diego Kauffmann-Guerrero, Marcus Unterrainer, Peter Bartenstein, Amanda Tufman

**Affiliations:** 1 Department of Nuclear Medicine, University Hospital, LMU Munich, Marchioninistr. 15, 81377 Munich, Germany; 2 DIE RADIOLOGIE, Munich, Germany; 3 Division of Respiratory Medicine and Thoracic Oncology, Department of Internal Medicine V, Thoracic Oncology Center Munich, University Hospital, LMU Munich, Munich, Germany; 4 Department of Radiology, University Hospital, LMU Munich, Munich, Germany

The role of c-MET as member of the receptor tyrosine kinase superfamily in the signaling pathway of non-small cell lung cancer (NSCLC) has been extensively described [1, 2]. MET is overexpressed in up to 60% of NSCLC cases, and in tumors harboring MET mutations this molecular structure is directly targeted using tyrosine kinase inhibitors (TKI) for therapeutic purposes [3].

^68^Ga-EMP-100 is a novel PET ligand that addresses the tyrosine kinase c-MET and has already shown its applicability in patients with metastatic renal cell carcinoma [4].

We present a case of a 56-year-old female former smoker with stage IV EGFR mutation-positive NSCLC (lymphogenic, pulmonary, hepatic, and cerebral metastases. Stage: cT3, cN3, cM1b) and concomitant MET exon 14 skipping mutation. After multiple pretreatments including radiochemotherapy and additional systemic therapies with afatinib, crizotinib, osimertinib, and cabozantinib, the patient underwent ^18^F-FDG PET/CT for treatment monitoring purposes, where multiple pulmonary and hepatic metastases with high glucose consumption were noted (Fig. 1A, B). Moreover, the patient underwent ^68^Ga-EMP-100 PET/CT (6 days before ^18^F-FDG PET/CT) to further assess c-MET expression in these tumoral sites. Here, a highly heterogeneous c-MET expression was found with some lesions showing high c-MET expression, but also lesions with only slight or even barely any increased c-MET expression despite their high glucose consumption on ^18^F-FDG PET (see Fig. 1 A vs. C and Fig. 1 B vs. D). Overall, elevated tumor metabolism was not necessarily accompanied by high c-MET expression.

This case illustrates the heterogeneity of tumor molecular biology in the stage IV setting, and the potential of ^68^Ga-EMP-100 PET/CT for pre-therapeutic and non-invasive assessment of whole-body c-MET expression in NSCLC patients prior to c-MET targeting tyrosine kinase inhibitors such as cabozantinib [2, 5]. Tissue biopsies may underestimate the heterogeneity of tumor lesions. Molecular imaging may provide a broader assessment of the distribution and relevance of particular molecular features, such as the c-MET expression as assessed with ^68^Ga-EMP-100 PET/CT, and thus inform treatment priorities.

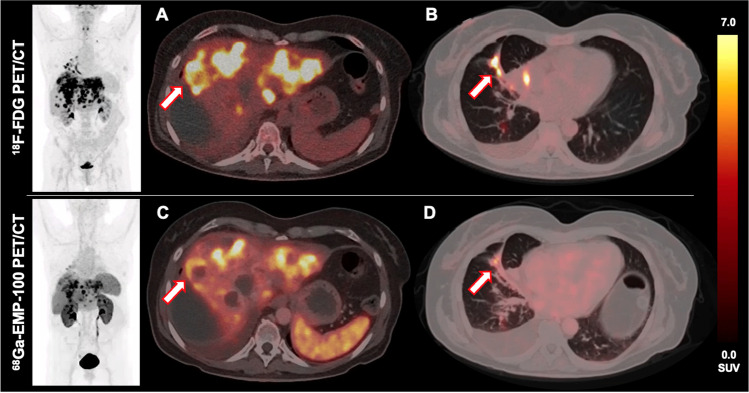



## Declarations

This analysis was performed in compliance with the principles of the Declaration of Helsinki and its subsequent amendments. The patient’s relatives have given their consent to the publication of this data. The publication of these data was approved by the institutional ethics board of the LMU Munich. Lena Maria Unterrainer was supported by the Clinician Scientist Program of the LMU Munich.
